# Naturally Occurring N-Terminal Fragments of Bovine Milk Osteopontin Are Transported across Models of the Intestinal Barrier

**DOI:** 10.3390/biomedicines11030893

**Published:** 2023-03-14

**Authors:** Brian Christensen, Nanna R. Nielsen, Marie R. Sørensen, Lotte N. Jacobsen, Marie S. Ostenfeld, Esben S. Sørensen

**Affiliations:** 1 Department of Molecular Biology and Genetics, Aarhus University, DK-8000 Aarhus, Denmark; bc@mbg.au.dk (B.C.);; 2 Arla Foods Ingredients Group P/S, DK-8260 Viby J, Denmark; lojan@arlafoods.com (L.N.J.); mstos@arlafoods.com (M.S.O.)

**Keywords:** osteopontin, intestinal cells, Caco-2, HT29-MTX, protein transport, gastrointestinal digestion

## Abstract

Osteopontin (OPN) is a bioactive integrin-binding protein found in high concentrations in milk, where it is present both as a full-length protein and as several N-terminally derived fragments. OPN resists gastric digestion, and via interaction with receptors in the gut or by crossing the intestinal barrier into circulation, ingested milk OPN may influence physiological processes. The aim of this study was to investigate OPN interaction with intestinal cells and its transport across models of the intestinal barrier. Immunodetection of OPN incubated with Caco-2 cells at 4 °C and 37 °C showed that OPN binds to the intestinal cells, but it is not internalised. Transepithelial transport was studied using mono- and co-cultures of Caco-2 cells and mucus-producing HT29-MTX cells in transwell membranes. OPN was shown to cross the barrier models in a time-, temperature-, and energy-dependent process inhibited by wortmannin, indicating that the transport takes place via the transcytosis pathway. Analyses of the naturally occurring milk mixture of full-length and N-terminal fragments showed that the N-terminal fragments of OPN bound intestinal cells most effectively and that the fragments were transported across the intestinal membrane models. This suggests that proteolytic processing of OPN increases its biological activity after ingestion.

## 1. Introduction

Milk is a rich source of bioactive molecules with the potential to initiate and support numerous physiological processes in neonates and growing infants. For example, the milk proteins lactoferrin, α-lactalbumin, and casein possess antimicrobial activities and participate in the development and maturation of the intestinal tissue, immunological processes, and transportation of essential minerals [1,2]. Another bioactive milk protein is osteopontin (OPN), which is among the 10 most abundant proteins in human milk [3] with a concentration of 99–266 mg/L [4]. In comparison, pooled bovine dairy milk and infant formula have been reported to contain concentrations around 18 mg/L and 9 mg/mL, respectively [5].

OPN is an acidic and phosphorylated glycoprotein containing the Arg-Gly-Asp (RGD) integrin-binding sequence and a second cryptic integrin-binding motif (SVAYGLK in bovine OPN) located next to the RGD sequence [6]. The amino acid sequences of bovine and human OPN are very similar, and integrin-binding sites as well as motifs for phosphorylation and glycosylation are conserved among the two species [7]. In human and bovine milk, OPN is present as an intact full-length protein and as a group of N-terminal-derived fragments resulting from endogenous proteolytic activity in the milk (Figure S1A) [8,9].

Milk OPN resists digestion by neonatal gastric aspirates [10], and a bovine milk OPN fragment containing the integrin-binding motifs is protected against pepsin digestion by glycosylations near the RGD sequence [11]. Therefore, milk OPN or fragments thereof containing the integrin-binding motifs are expected to reach the duodenum in a form where it can potentially induce physiological effects in the gut. In support hereof, several studies have shown that ingestion of bovine milk OPN influences physiological processes. A large clinical trial showed that infants receiving formula fortified with bovine milk OPN had fewer days with fever and significantly reduced levels of pro-inflammatory cytokines compared to infants receiving non-supplemented formula [12]. In another study, the intestinal transcriptome of new-born rhesus monkeys was changed significantly by formula supplemented with bovine milk OPN [13]. Likewise, it was recently shown that the transcriptome of Caco-2 cells was significantly affected when exposed to digested human and bovine OPN [14]. In pig and mouse models, supplementation with bovine milk OPN increased villus height and crypt depth in the small intestine compared to controls [15,16]. Intestinal absorption of dietary bovine OPN has been indicated, as bovine milk OPN species have been detected in the plasma of infants fed formula supplemented with bovine milk OPN [17] and in mice fed bovine milk OPN by gavage [18,19]. Bovine milk OPN has also been shown to cross the blood-brain barrier in mouse pups, where it increases the expression of proteins related to myelination and improves learning abilities [15,20].

Collectively, these studies suggest that ingested milk OPN, OPN fragments, or both can mediate physiological effects via interactions in the small intestine or after transport across the intestinal barrier to the circulation. However, in these studies, OPN has been detected by immunohistochemistry, competitive ELISA, or tracing of radioactivity, and it has not been shown which forms or fragments of OPN were absorbed in the intestinal barrier and hence were responsible for the biological effects reported. In the present study, we used human intestinal cells to investigate the binding, uptake, and transport of full-length OPN and N-terminal fragments of OPN naturally present in bovine milk. The specific aim was to determine which forms of OPN interact with the intestinal cells and are subsequently transported across the intestinal barrier and to describe the mechanism of this transport. By incubation with Caco-2 cells, it was shown that OPN binds to the intestinal cells but is not internalised. Apical-to-basolateral transport assays using Caco-2 cells and mucus-producing HT29-MTX cells revealed that OPN crosses the Caco-2 intestinal barrier model via the transcytosis pathway. Interestingly, the N-terminal OPN fragments interact with intestinal cells and are transported across the intestinal epithelium more effectively than the full-length protein.

## 2. Materials and Methods

### 2.1. Chemicals

Gly-Sar, cytochalasin D, wortmannin, RIPA lysis buffer, bovine serum albumin, N-hydroxysuccinimide biotin, pepsin from porcine gastric mucosa, and ovalbumin were from Merck (Rødovre, Denmark). Trypsin, chymotrypsin, and elastase (all from bovine pancreas) were from Worthington Biochemical Corporation (Lakewood, NJ, USA).

### 2.2. Proteins and Antibodies

Bovine milk OPN (Lacprodan^®^ OPN-10) was obtained from Arla Foods Ingredients (Viby J, Denmark). Lacprodan^®^ OPN-10 contains ~78% protein, of which 95% is OPN. The full-length OPN and the N-terminal OPN fragments were separated by gel filtration on a Superdex 200 HR 10/30 column (GE Healthcare, Uppsala, Sweden) connected to a GE Healthcare LKB system as described [8]. Proteose peptone component 3 (PP3) was purified from bovine milk as described [21].

Polyclonal antibodies against OPN and PP3 were raised in rabbits by DAKO A/S (Glostrup, Denmark) as described [10]. The monoclonal antibody MAB193p that recognises an epitope located in the N-terminal part of OPN was from BBI Solutions (Portland, ME, USA). For biotinylation, antibodies were dialysed against 0.1 M sodium borate buffer, pH 8.8, overnight at 4 °C. One-sixth (*v*/*v*) of 10 mg/mL of N-hydroxysuccinimide biotin was added to the antibodies. After 4 h of incubation at room temperature, the reaction was stopped with 1 M of ammonium bicarbonate per 250 μg of N-hydroxysuccinimide biotin. Finally, the biotinylated antibodies were dialysed against phosphate-buffered saline (PBS) overnight at 4 °C as described [5].

### 2.3. In Vitro Gastrointestinal Digestion

Bovine milk OPN was subjected to digestion essentially as described in [22,23] with few modifications. The protein was dissolved in 0.15 M NaCl, pH 2.5, at a concentration of 1 mg/mL and incubated with pepsin in a 1:50 w/w enzyme-to-substrate ratio for 60 min at 37 °C. Before digestion with pancreatic proteases, the sample was lyophilised and washed twice in deionised water. Trypsin and chymotrypsin (1:50 *w*/*w*) and elastase (1:250 *w*/*w*) were added to 50 mM ammonium bicarbonate buffer, and the mixture was incubated for 60 min at 37 °C. The proteases were then inactivated by adding phenylmethanesulfonyl fluoride to 1.5 mM.

### 2.4. Cell Cultures

The human colorectal adenocarcinoma cell line Caco-2 was obtained from DSMZ (Braunschweig, Germany), and HT29-MTX-E12 cells were from Merck (Rødovre, Denmark). The cells were grown to 70–80% confluence in Dulbecco’s Modified Eagle’s Medium (DMEM) with Glutamax supplemented with 10% heat-inactivated fetal bovine serum, 100 units/mL penicillin, and 100 μg/mL streptomycin (all from Invitrogen, Carlsbad, CA, USA) in a humidified 5% CO_2_/95% air atmosphere at 37 °C. The Caco-2 cells were grown alone or together with different proportions of HT29-MTX cells. For experiments, Caco-2 and HT29-MTX cells were in passages 10–30 and 53–63, respectively.

### 2.5. Cell Binding Assays

The cells were seeded in tissue culture-treated polystyrene plates (Corning Inc., Corning, NY, USA) (75,000 cells/cm^2^). After differentiation for 15 days, the Caco-2 cells were fasted with serum-free medium (SFM) for 30 min, and then the cells were incubated with OPN or PP3 (10, 50, or 100 μg/mL) in SFM at 37 °C for 2 h. A time series was conducted by incubating cells with 50 μg/mL OPN for 5, 15, 30, 60, 90, or 120 min. The influence of temperature was investigated by comparing cell treatment at 37 °C to treatment at 4 °C [24]. Cells were pre-incubated for 2 h at 4 °C, followed by incubation with OPN for 2 h at 4 °C. For analysis of OPN internalisation, the cells were incubated with OPN for 2 h at 37 °C, washed twice with PBS, and then 0.05% trypsin was added for 3 min in order to degrade all non-internalised or membrane-bound proteins as described [25]. In all experiments, incubation with SFM was used as a negative control. After incubation, the cells were washed four times with a 10 mM sodium citrate buffer containing 0.05% (*v*/*v*) Tween 20, pH 6, as described [26], and then lysed with RIPA lysis buffer. The cell lysate was collected and centrifuged for 10 min at 9000× *g* at 4 °C to separate the supernatant. The protein concentration in the lysates was measured using a micro BCA protein assay kit (Thermo Fisher Scientific, Hillsboro, OR, USA) according to the manufacturer’s protocol. OPN and PP3 were affinity purified using Dynabeads M-280 Tosyl-activated (Invitrogen, Carlsbad, CA, USA) coupled with polyclonal antibodies directed against the target proteins according to the manufacturer’s procedure. To isolate the target proteins, 20 μL cell lysate was mixed with 0.25 mg antibody-coupled Dynabeads in PBS containing 0.1% BSA and incubated for 1 h at room temperature. The beads were washed three times with PBS, dissolved in 25 μL PBS, and incubated for 3 min at 95 °C to elute the proteins from the beads. OPN and PP3 were detected by Western blotting as described below. One hundred nanograms of OPN or PP3 was used as a positive control in the purification with Dynabeads.

### 2.6. Transepithelial Transport Studies

The cells were seeded in polyethylene terephthalate culture inserts with a pore size of 0.4 µm and a growth area of 0.33 cm^2^, at a density of approximately 75,000 cells/cm^2^. The cells were grown on the transwell membrane for three weeks before they were used for transport studies. For transport experiments, the integrity of the monolayers was evaluated by measuring the transepithelial electrical resistance (TEER) using a Millicell ERS-2 volt-ohmmeter (Merck, Rødovre, Denmark). The TEER was measured before and at the end of the experiment, and as recommended, only Caco-2 monolayers with TEER-values of 250–300 Ω cm^2^ were used [27].

Following the TEER measurement, the cells were gently washed twice with PBS, and SFM was added to the apical and basolateral compartments. For inhibition experiments, cells were preincubated for 30 min with Gly-Sar (10 mM), cytochalasin D (0.5 µg/mL), wortmannin (0.5 or 1 µM), or NaN_3_ (10 mM) before OPN or PP3 (both at 50 µg/mL) with or without the presence of inhibitors were added to the apical compartments and incubated for 2 h [27]. Temperature dependency was evaluated by preincubating the cells for 2 h at 4 °C and conducting the transport assay at 4 °C as well. After 0.5 h, 1 h, 1.5 h, and 2 h, apical and basolateral medium was collected. Apical (0.5 μL) and basolateral (200 μL after ethanol precipitation) medium was analysed by Western blotting.

Membrane integrity was further validated by the addition of D-[1-14C]-Mannitol (Perkin Elmer, Waltham, MA, USA) to the upper chamber of culture inserts with or without milk proteins for 2 h. Basolateral samples were counted in a scintillation counter, and the apparent permeability coefficient (Papp, cm/s) was determined as Papp = (dQ/dt)/(AC0) where dQ/dt is the steady state flux (cpm/s), A is the surface area of the cell culture insert membrane, and C0 is the initial concentration in the apical compartment (cpm/mL) as described in [28].

### 2.7. MTT Assay

An MTT (3-(4,5-dimethylthiazol-2-yl)-2,5-diphenyltetrazolium bromide) test was performed following a standard protocol [26]. Caco-2 cells were seeded in a flat-bottom 96-well tissue culture-treated microplate (Costar 3628, Thermo Fisher Scientific, Hillsboro, OR, USA) at a density of 12,000 cells/well and grown for three days. Thereafter, the medium was replaced with SFM containing OPN at different concentrations (50, 100, and 200 μg/mL) for 2 h. The MTT assay was conducted according to Thermo Fisher Scientific’s protocol (Vybrant^®^ MTT Cell Proliferation Assay Kit, Thermo Fisher Scientific, Hillsboro, OR, USA). Briefly, the cells were washed once in PBS and then incubated in phenol red-free DMEM containing 1 mM MTT solution. After 2 h, the media was discarded, and formazan crystals were solubilised with dimethyl sulfoxide. The amount of metabolically active cells was quantified by measuring the absorbance at 540 nm (EnSpire Multimode Plate Reader; Perkin Elmer, MA, USA) and compared to the control.

### 2.8. ELISA of Cell Lysates

An in-house developed enzyme-linked immunosorbent assay (ELISA) [29] was used to quantify the levels of OPN in cell lysates. A 96-well MaxiSorp immunoassay plate (Thermo Fisher Scientific, Hillsboro, OR, USA) was coated with 0.5 μg/mL bovine milk OPN IgG in 0.1 M sodium carbonate, pH 9.8, overnight at 4 °C, washed with PBS, and blocked with 2% (*w*/*v*) ovalbumin in PBS for 1 h at 37 °C. Concurrently, cell lysates and OPN standard samples were diluted in PBS with 0.1% (*v*/*v*) Tween 20 (PBS-T) and incubated with 2.5 μg/mL biotinylated MAB193p for 1 h at 37 °C. After washing the plate with PBS-T, the pre-incubated cell lysates and OPN standard samples were applied in duplicate and incubated for another hour at 37 °C, followed by washing. Detection of captured OPN was performed by incubation with streptavidin-HRP conjugate (GE Healthcare, Uppsala, Sweden) diluted 1:10,000 (*v*/*v*) in PBS-T for 1 h at 37 °C. After washing, colour development was obtained from reaction with TMB-ONE that was subsequently quenched with 0.2 M sulphuric acid, and absorbance at 450 nm was measured in a microplate reader. The OPN content in cell lysates was calculated from the standards and normalised to total cell protein.

### 2.9. Western Blotting

Samples from Dynabeads or transport experiments were separated on 10% NuPAGE Novex bis-Tris precast gels (Invitrogen, Carlsbad, CA, USA), followed by electroblotting onto Hybond-P PVDF membranes (GE Healthcare, Uppsala, Sweden) for immunodetection. The membranes were blocked with 2% Tween in Tris-buffered saline before the addition of the polyclonal rabbit OPN or PP3 antibodies (5 μg/mL) (the antibodies were biotinylated for detection of proteins eluted from the Dynabeads). OPN and PP3 were detected with HRP-conjugated secondary immunoglobulins or with streptavidin-HRP conjugate using enhanced chemiluminescence on an ImageQuant LAS 4000 instrument (GE Healthcare, Uppsala, Sweden).

### 2.10. Statistical Analysis

All data presented are the means ± standard deviation (SD). The values are compared using one-way ANOVA with Dunnett’s or Tukey’s multiple comparisons test or two-way ANOVA with the Bonferroni post hoc test. Model validation included tests for equal standard deviations and normal distribution. Statistical analyses were performed using GraphPad Prim software (version 9.5.1, San Diego, CA, USA). Differences were considered significant at *p* < 0.05.

## 3. Results

### 3.1. Dose-Response and Time-Dependent Binding of Osteopontin to Caco-2 Cells

**Figure 1:**
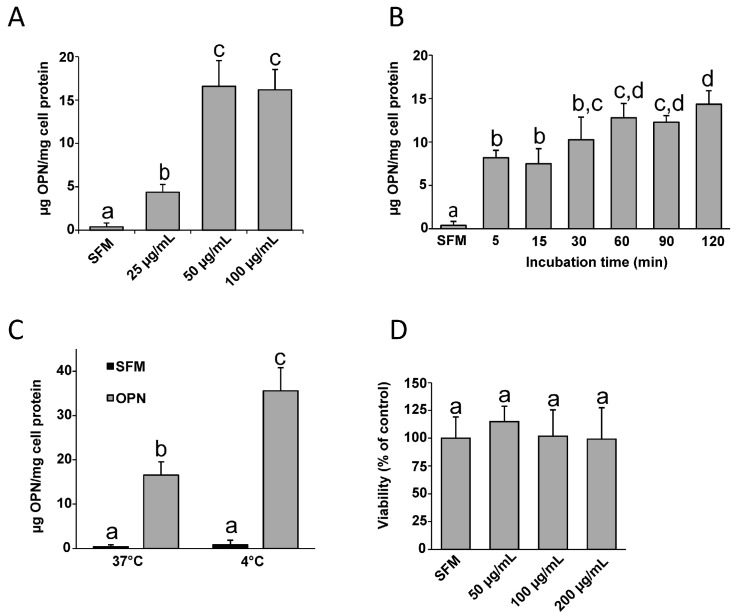
ELISA of intestinal Caco-2 cell lysates incubated with osteopontin (OPN). Caco-2 monolayers were incubated with serum-free medium (SFM) or OPN, washed extensively, and lysed. The OPN content in lysates was quantified by ELISA and normalised to the total protein concentration in the lysates. (**A**) Cells incubated with OPN at the indicated concentration for 2 h. (**B**) Cells incubated with 50 µg/mL OPN for the indicated times. (**C**) Cells incubated with SFM or OPN for 2 h at 4 °C or 37 °C. In (**A**–**C**), data are expressed as mean ± SD (n = 4) and the experiments were repeated two to three times. (**D**) Cytotoxicity of OPN was evaluated using an MTT assay. Cells were incubated with OPN (50, 100, or 200 μg/mL) or SFM for 2 h. Data are expressed as mean ± SD (n = 10) and are representative of three individual experiments. Statistical analysis: one-way ANOVA followed by Dunnett’s test (**A**,**D**), one-way ANOVA followed by Tukey’s test (**B**), or two-way ANOVA (**C**) followed by Bonferroni’s test. Different letters indicate significant differences, *p <* 0.05.

The OPN used in this study was purified from bovine milk and consists of a naturally occurring mix of the intact full-length protein and N-terminal OPN fragments resulting from endogenous proteolysis in the milk (Figure S1) [8]. The bovine milk OPN was incubated with Caco-2 cells to evaluate whether the protein interacts with intestinal cell membranes. Following incubation, the cells were washed extensively with citrate buffer and lysed, and then OPN was identified by ELISA. OPN was detected in lysates from cells incubated with 25 µg/mL OPN, but not in lysates of cells incubated with only cell culture medium (Figure 1A). The quantity of OPN interacting with cells increased about three-fold after incubation of the cells with 50 μg/mL OPN, while the addition of higher concentrations of OPN did not increase the quantity of OPN in the lysates further. Next, the interaction between OPN and the cells as a function of incubation time was investigated. OPN was detected 5 min after the addition of the protein to the Caco-2 cells, and the quantity of OPN interacting with the cells did not increase further after incubation for 1 h (Figure 1B).

Generally, the internalisation of proteins in mammalian cells ceases when the temperature is lower than 10 °C [30], hence the Caco-2 cells were incubated with OPN at 4 °C or 37 °C to investigate whether the OPN measured in the lysates was a result of extracellular binding or internalisation. OPN was detected from cells incubated with the protein at 4 °C and at 37 °C, and the quantity of OPN associated with the lysates was two- to three-fold higher at 4 °C than at 37 °C (Figure 1C). To further explore if the OPN was membrane-bound, the cells at 37 °C were washed with PBS and incubated with 0.05% trypsin or PBS (as a control) for 3 min. Western blotting showed that the trypsin treatment removed OPN from the lysate, confirming that at 37 °C there is little, if any, cellular uptake of OPN (Figure S2).

An MTT assay was used to analyse whether OPN displayed cytotoxicity towards the Caco-2 cells with increasing concentration. OPN did not cause cytotoxicity when compared to cells not exposed to OPN (Figure 1D).

### 3.2. Binding of Full-Length Osteopontin and N-Terminal Fragments to Caco-2 Cells

**Figure 2:**
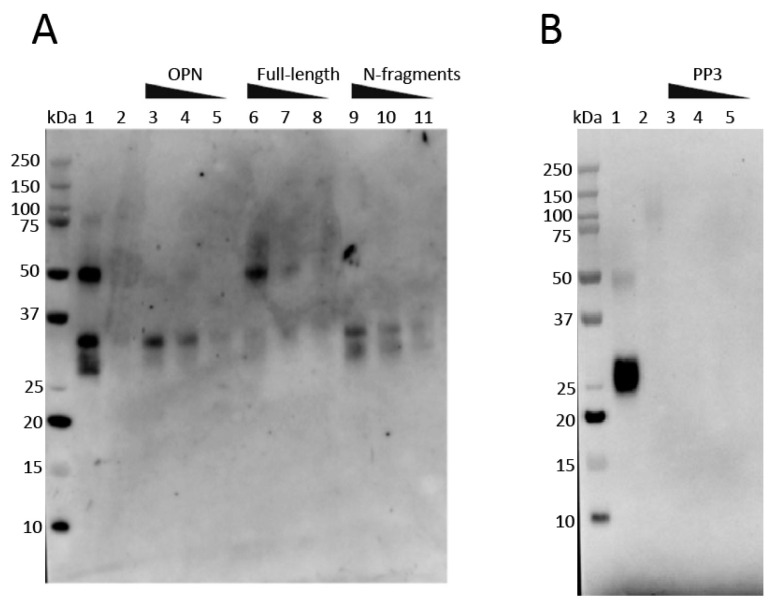
Western blotting of Caco-2 cell lysates after incubation with osteopontin (OPN), full-length OPN, or N-terminal OPN fragments separated by gel filtration. Cell lysates were incubated with anti-OPN IgG-coupled Dynabeads before Western blotting, and OPN was detected using the biotinylated anti-OPN antibody MAB193p. (**A**) Lane 1, 100 ng OPN (positive control for purification); lane 2, cells incubated without OPN; lanes 3–5, cells incubated with OPN (lane 3, 100 μg/mL; lane 4, 50 μg/mL; lane 5, 10 μg/mL); lanes 6–8, cells incubated with full-length OPN (lane 6, 100 μg/mL; lane 7, 50 μg/mL; lane 8, 10 μg/mL); lanes 9–11, cells incubated with N-terminal OPN fragments (lane 9, 100 μg/mL; lane 10, 50 μg/mL; lane 11, 10 μg/mL). (**B**) Cell lysates incubated with PP3. PP3 was purified from lysates with anti-PP3 IgG-coupled Dynabeads before Western blotting. Lane 1, 100 ng PP3; lane 2; cells incubated without PP3; lanes 3–5, cells incubated with PP3 (lane 3, 100 μg/mL; lane 4, 50 μg/mL; lane 5, 10 μg/mL). Both blots are representative of three individual experiments.

To analyse which forms of OPN interact with the Caco-2 cells, the cell lysates were subjected to Western blotting after affinity purification of OPN using OPN IgG-conjugated Dynabeads. As a control experiment for the affinity purification, the OPN preparation supplemented to the cells was analysed, and the binding of both the N-terminal OPN fragments and the full-length protein migrating at 35 and 50 kDa, respectively, was shown (Figure 2A, lane 1). The N-terminal fragments, but not the full-length protein, were observed in the cell lysates after a 2 h incubation with bovine milk OPN that contains a mix of full-length OPN and the N-terminal fragments (Figure 2A, lanes 3–4). Full-length OPN and the N-terminal fragments were separated by gel filtration (Figure S1), and the separated components were incubated with the Caco-2 cells. Both the full-length protein (Figure 2A, lanes 6–8) and the fragments (Figure 2A, lanes 9–11) were detected in the cell lysates when they were applied to the cells separately. As a protein control, the cells were incubated with another milk protein, PP3, and the resulting lysates were subjected to Western blotting. PP3 was not detected in lysates from cell monolayers incubated with 10, 50, and 100 ug/mL (Figure 2B, lanes 3–5).

### 3.3. Apical-to-Basolateral Transport of Osteopontin over Caco-2 Cell Monolayer

**Figure 3:**
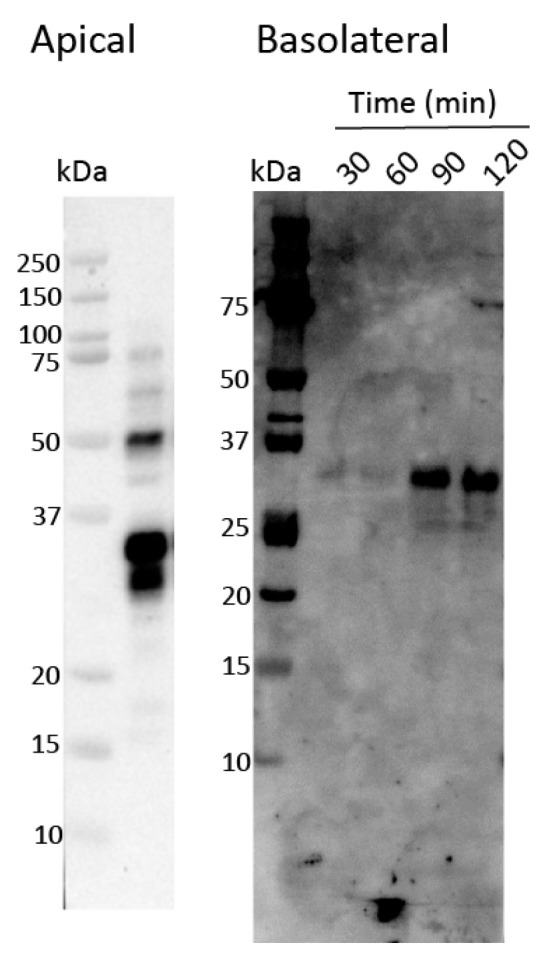
Epithelial transport of osteopontin (OPN). Caco-2 cells were grown on transwell membranes and incubated with OPN in the apical compartment representing the intestinal luminal side. Samples from apical and basolateral compartments were collected separately after the indicated times, and OPN was detected in the medium by Western blotting using polyclonal anti-OPN antibodies. The Western blot of the apical sample is from medium collected after 2 h incubation with OPN, and it is representative of all incubation times. The blots are representative of three individual experiments.

In order to investigate whether OPN can cross an in vitro model of the intestinal barrier, OPN was added to the apical side of differentiated Caco-2 monolayers (representing the intestinal luminal side). After 0.5 h, 1 h, 1.5 h, and 2 h, the transcellular transport was monitored by Western blot analysis of the basolateral media (representing the blood side). In the apical compartment, both full-length and N-terminal fragments of OPN were observed (Figure 3). In the basolateral compartment, OPN was not detected after 30 min and was only faintly observed after 1 h, but bands representing the N-terminal fragments migrating at ~35 kDa were clearly present after 1.5 h (Figure 3). Full-length OPN was only detected in the apical, and not in the basolateral, compartment.

### 3.4. Analysis of the Mechanism of Osteopontin Transport

**Figure 4:**
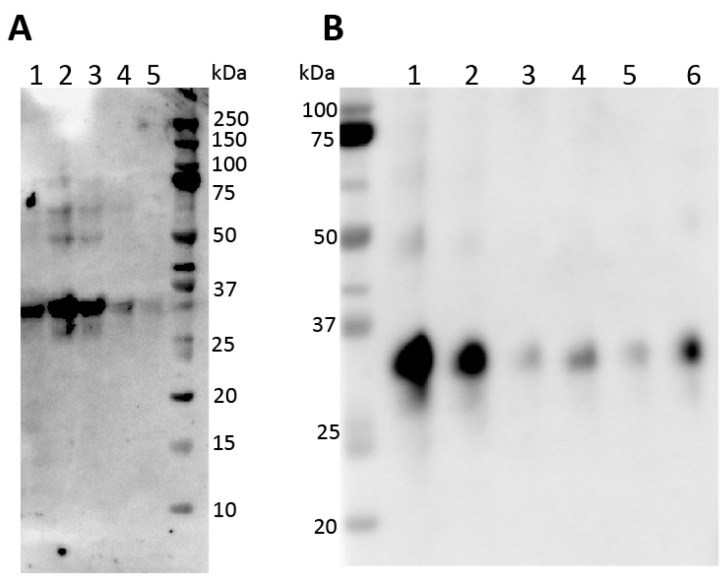
Inhibition of epithelial transport of osteopontin (OPN). Caco-2 cells were grown on transwell membranes and incubated with OPN in the apical compartment representing the intestinal luminal side. The cells were pre-incubated with inhibitor solutions or incubated at 4 °C for 2 h before OPN was added, and then incubated for 2 h in the presence of inhibitors. OPN was detected in the basolateral medium by Western blotting using polyclonal anti-OPN antibodies. (**A**) Lane 1, cells incubated with OPN; lanes 2–5, cells incubated with OPN in the presence of inhibitors (lane 2, 10 mM Gly-Sar; lane 3, 0.5 μg/mL Cytochalasin D; lanes 4–5, 1 μM and 0.5 μM wortmannin. (**B**) Lanes 1–2, cells incubated with OPN at 37 °C; lanes 3–4; cells incubated with OPN at 37 °C in the presence of 10 mM NaN_3_; lanes 5–6, cells incubated with OPN at 4 °C. The blots are representative of three (**A**) or two (**B**) individual experiments.

To evaluate the pathway of the epithelial transport of OPN, the effect of inhibitors was evaluated. Based on intensity in Western blotting, the transport was unaffected by Gly-Sar, a competitive inhibitor of peptide transporter PepT1, and by cytochalasin D, a tight junction disruptor (Figure 4A). In contrast, wortmannin, an inhibitor of transcytosis, inhibited the transport of OPN (Figure 4A). Furthermore, OPN was added to the Caco-2 cell monolayers in the presence of sodium azide to investigate if the transport was an energy-dependent process. In the basolateral samples, the intensities of the ~35 kDa band representing N-terminal OPN fragments were lower in samples from cells treated with sodium azide compared to the controls, indicating that it was an energy-dependent process (Figure 4B). The transepithelial transport was also reduced at 4 °C compared to 37 °C, as evident from the lower intensity of the bands in samples from cells incubated at 4 °C compared to controls (Figure 4B).

### 3.5. Apical-to-Basolateral Transport of Osteopontin over Caco-2/HT29-MTX Co-Cultures

**Figure 5:**
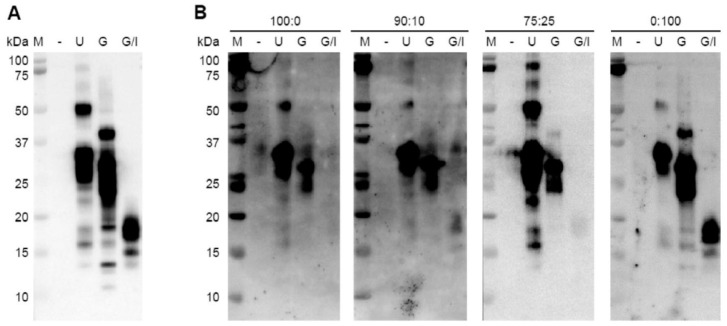
Epithelial transport of osteopontin (OPN) across Caco-2/HT29-MTX monolayers after simulated gastrointestinal digestion. Cells were seeded at different Caco-2:HT29-MTX proportions on transwell membranes. Undigested OPN (U) and OPN after simulated gastric digestion (with pepsin (G)) or gastrointestinal digestion (with pepsin and subsequently trypsin, chymotrypsin, and elastase (G/I)) was added to the apical compartment of the monolayers. Cells only added serum-free medium (-) were used as control. After 2 h, apical and basolateral medium was analysed by Western blotting using polyclonal anti-OPN IgG. (**A**) Apical samples are representative for all Caco-2:HT29-MTX proportions. (**B**) Basolateral samples from the different cell cultures. The Caco-2:HT29-MTX seeding proportions are given above the gels. The blots are representative of two individual experiments.

Mucus-secreting HT29-MTX and co-cultures of Caco-2 and HT29-MTX cells were grown to investigate the transcellular transport of OPN in a more in vivo-relevant setup. To simulate gastrointestinal transit, OPN was digested with pepsin and then with trypsin, chymotrypsin, and elastase before the protein digest was applied to the different mono- and co-cultures. OPN subjected to simulated gastrointestinal digestion did not cause cytotoxicity when compared to cells not exposed to the protein (Figure S3). OPN and pepsin-digested OPN were observed in the basolateral compartment of all Caco-2 and HT29-MTX mono- and co-cultures (Figure 5). The OPN fragment resulting from combined gastric and intestinal digestion was only observed in the HT29-MTX monoculture (Figure 5B).

**Figure 6:**
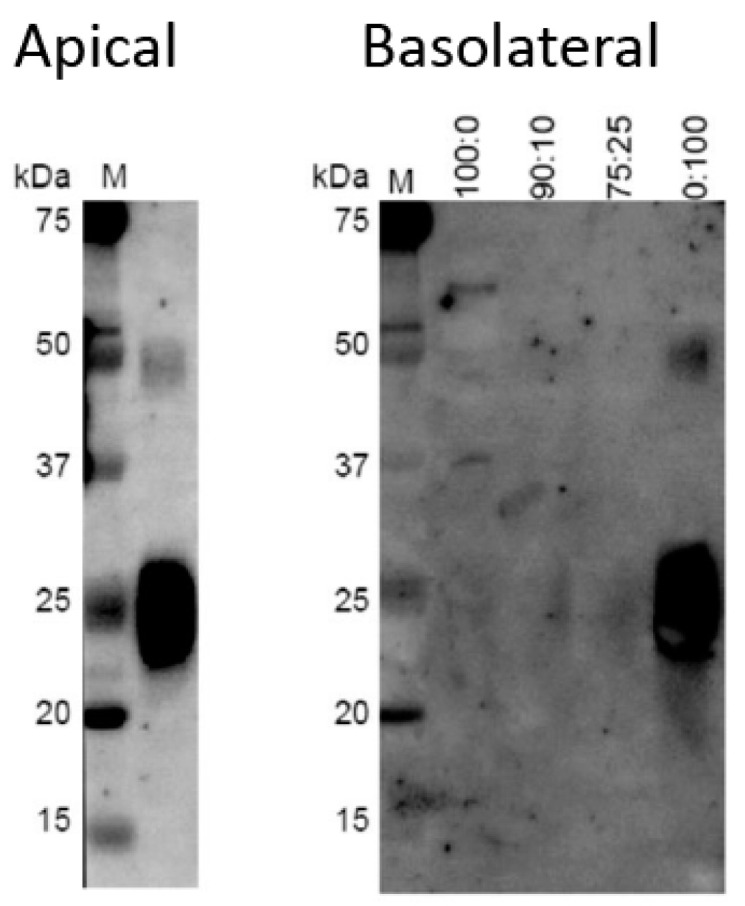
Epithelial transport of PP3 across Caco-2/HT29-MTX monolayers. Cells were grown at different Caco-2:HT29-MTX proportions on transwell membranes. PP3 was added to the apical compartment of the monolayers, and after 2 h, apical and basolateral medium was analysed by Western blotting using polyclonal anti-PP3 IgG. The Western blot of the apical sample is representative of all Caco-2:HT29-MTX proportions. For basolateral samples, the Caco-2:HT29-MTX proportions are given above the gels. The blots are representative of two individual experiments.

The control protein PP3 was only detected in the basolateral sample from the HT29-MTX monoculture (Figure 6). The HT29-MTX monoculture had lower TEER and higher Papp values than the Caco-2 monolayer and the Caco-2:HT29-MTX co-cultures. The TEER of ~300 Ω cm^2^ indicates that the monolayers of Caco-2 cells alone and of Caco-2:HT29-MTX co-cultures fulfil the requirements for use in transport experiments [31]. The TEER values did not decrease during the transport assay (Figure S4).

## 4. Discussion

The well-established human Caco-2 cell line was used to investigate the interaction and transepithelial transport of bovine milk OPN across intestinal enterocytes. The Caco-2 cells differentiate in culture to form a confluent monolayer of polarised cells with morphological and functional traits similar to mature enterocytes [32,33]. E.g., Caco-2 monolayers display microvilli, transport systems, and tight junctions between neighbouring cells. In a previous study, we have shown that the apparent permeability, TEER, and expression of tight junction proteins indicate that the cells form a monolayer that can be used as an in vitro model of the intestinal epithelium [28]. OPN, either before or after simulated gastrointestinal digestion, showed no negative effects on cell viability, as validated in MTT assays. This was expected, as bovine milk does not show any adverse effects in mice, rats, or human cell lines [34]. Likewise, no adverse effects were observed in infant rhesus monkeys or human infants [12,13].

To analyse the interaction of OPN with Caco-2 cells, the cells were incubated with the protein and then washed extensively with a citrate buffer before lysis. This washing step effectively removes unspecifically bound proteins from cell surfaces [26] before the detection of OPN in the cell lysate. OPN was detected in the lysate of Caco-2 cells after 5 min of incubation, which indicates a rapid interaction with the cells that seemed to saturate after 30–60 min. The OPN was purified from bovine milk, where it exists as a mixture of the full-length protein and N-terminal fragments [7,8]. Interestingly, only the N-terminal OPN fragments, and not the full-length protein, were present in the lysate from Caco-2 cells. This indicates that the N-terminal fragments have a stronger affinity for the cells than the full-length protein. However, the full-length protein was also observed after the incubation of Caco-2 cells with purified full-length OPN. As a control for the specificity of the observed interaction, the Caco-2 cells were incubated with the milk protein PP3, another acidic and phosphorylated glycoprotein from bovine milk [21]. PP3 was not detected in the cell lysates, showing that the interaction is not an unspecific protein interaction, but specific for OPN.

The OPN present in the Caco-2 lysates could originate either from OPN bound extracellularly to the cell membranes or from internalised OPN inside the cells. To distinguish between these options, the experiments were conducted at 4 °C and 37 °C, as it is well established that endocytosis does not take place at 4°C, whereas binding to cell surfaces may still occur [30]. OPN was detected at 4 °C, indicating that the protein was bound to the membranes rather than being internalised by endocytosis. To further verify that OPN is located extracellularly, trypsin was added to the cells after incubation at 37 °C for 2 h. If OPN had entered the cells, it would be inaccessible to trypsin added to the culture medium. The trypsin treatment confirmed that OPN had been bound to the cell surface as it was removed by trypsin treatment. Previously, OPN has been reported to bind to Caco-2 cells at 4 °C and to be internalised at 37 °C [24]. In our study, the amount of OPN bound to the membrane was higher at 4 °C than at 37 °C, and this difference could be caused by the internalisation of OPN at 37 °C, which was not detected by the antibodies used in this study.

The differentiated Caco-2 monolayers were used to study the apical-to-basolateral transport of OPN. The Western blots indicated that the N-terminal OPN fragments were more effectively transported to the basal chamber compared to the full-length protein. The absence of the control protein PP3 in the basolateral medium suggests that the transport of OPN across the Caco-2 monolayers is strictly regulated. So that only specific components such as, e.g., the N-terminal OPN fragments, are granted access to the basolateral side, representing the blood side in the model. The observed molecular weight in Western blots of the OPN fragments indicates that the fragments are not degraded in the cells but are internalised and transported intact across the Caco-2 cells. The transport was inhibited by the presence of wortmannin, indicating transcytosis as a potential route for transepithelial transport. Furthermore, the transport was reduced in the presence of sodium azide, an inhibitor of cellular energy production, and when the assay was performed at 4 °C. These findings support that the N-terminal fragments of OPN are transported across the membrane by energy-dependent transcytosis. Transcytosis of milk components across Caco-2 monolayers has previously been shown for a 17-residue immunomodulatory peptide from β-casein and for larger milk components such as bovine milk β-lactoglobulin and α-lactalbumin [35,36].

For the investigation of OPN transport in an experimental setting more similar to in vivo conditions, a co-culture of Caco-2 cells and the mucin-producing goblet cell line HT29-MTX was established. Inclusion of the mucus-producing cells makes the Caco-2/HT29-MTX culture a better model of the human intestinal barrier compared to the conventional Caco-2 monoculture. A ratio of 90% Caco-2/10% HT29-MTX or 75% Caco-2/25% HT29-MTX has been used to mimic the cellular architecture of the small intestine, where absorption of proteins, peptides, and amino acids takes place [31,37]. To simulate gastrointestinal transit, OPN was digested with pepsin and the intestinal proteases chymotrypsin, trypsin, and elastase. As previously demonstrated, a bioactive fragment comprising residues Trp^27^-Phe^151^ resists gastric digestion in a form that enables interaction with integrins [11]. In our study, we observed that undigested OPN and an N-terminal fragment that is likely to constitute the Trp^27^-Phe^151^ fragment were transported to the basal side of the different co-culture monolayers. In contrast, the OPN fragment resulting from gastric and intestinal digestion and the control protein PP3 were only observed in the basolateral compartment of the HT29-MTX monoculture. This shows that OPN and the fragment resisting peptic digestion are actively and selectively transported across the Caco-2 and HT29-MTX co-culture monolayers and that their presence at the basal side of the intestinal models is not merely a result of diffusion or membrane leakage. OPN and PP3 observed in the basal compartment of the HT29-MTX monoculture are likely caused by passive diffusion, which is supported by the lower TEER and higher Papp values of the HT29-MTX monoculture compared to the co-cultures.

Uptake or transport of intact lactoferrin and ovalbumin across Caco-2 monolayers have previously been described [38,39]. In the present study, we observed that N-terminal fragments of OPN preferentially interacted with Caco-2 cell monolayers compared to the full-length protein, and the fragments were transported across different models of the intestinal barrier. In bovine milk, the N-terminal-derived fragments comprise residues 1–145/147/149/151/152/153, which expose the two integrin binding sequences RGD- and SVAYGLK in OPN. The remaining C-terminal fragment is presumed to be completely degraded by milk proteases [8]. Studies have shown that the N-terminal fragments of OPN bind the OPN receptors with a higher affinity than the intact OPN protein, presumably due to increased exposure of the integrin-binding motifs after cleavage [8,9]. These receptor-binding sites also resist gastric digestion in the stomach [11]. This is a prerequisite for the interaction with the α_4_β_1_ and α_9_β_1_ integrins [6], and the cleaved OPN is a much better ligand for α_5_β_1_ and α_v_β_3_ integrins compared to full-length OPN [9]. Caco-2 cells express α_4_,β_1_ and α_V_β_3_ [40], which potentially could participate in the binding and transport of OPN observed in this study. The enhanced ability to interact with cell surface integrins could potentially explain the abundance of the N-terminal fragments in the cell lysates and their presence in the basolateral medium.

## 5. Conclusions

This study investigated the interactions of bovine milk OPN with intestinal cells and its transport across a model of the intestinal barrier. It was demonstrated that OPN can bind Caco-2 cells and that the N-terminal fragments of OPN naturally present in milk bind more efficiently than the full-length protein. Likewise, OPN and especially the N-terminal fragments are transported across in vitro models of the intestinal barrier, both in samples of undigested OPN and after simulated gastric digestion. Transport was reduced at 4 °C compared to 37 °C by inhibition of cellular energy production and by wortmannin, which collectively indicates that OPN is transported across the intestinal barrier model by transcytosis. The transcytosis pathway is initiated by the internalisation of OPN at the apical membrane of enterocytes, which was not directly shown in this study. The results from the present study are in agreement with previous in vivo studies where bovine milk OPN species have been demonstrated to be present in plasma and tissues after ingestion.

## Data Availability

Not applicable.

## Supplementary Materials

The following supporting information can be downloaded at: https://www.mdpi.com/article/10.3390/biomedicines11030893/s1, Figure S1: Separation of full-length OPN and N-terminal fragments present in OPN purified from bovine milk; Figure S2: Evaluation of cell surface binding of OPN to Caco-2 cells by trypsin treatment. Figure S3: Cytotoxicity of OPN after simulated gastrointestinal digestion; Figure S4: Apparent permeability coefficient and TEER of Caco-2/HT29-MTX co-cultures.
