# Supplementary material for: Naturally Occurring N-Terminal Fragments of Bovine Milk Osteopontin Are Transported across Models of the Intestinal Barrier
Source: Biomedicines. 2023 Mar 14;11(3):893. doi: 10.3390/biomedicines11030893 (PMC10045268; doi:10.3390/biomedicines11030893)
Supplement: Supplementary file 1 [file biomedicines-11-00893-s001.zip › biomedicines-2210050 supplementary .pdf]

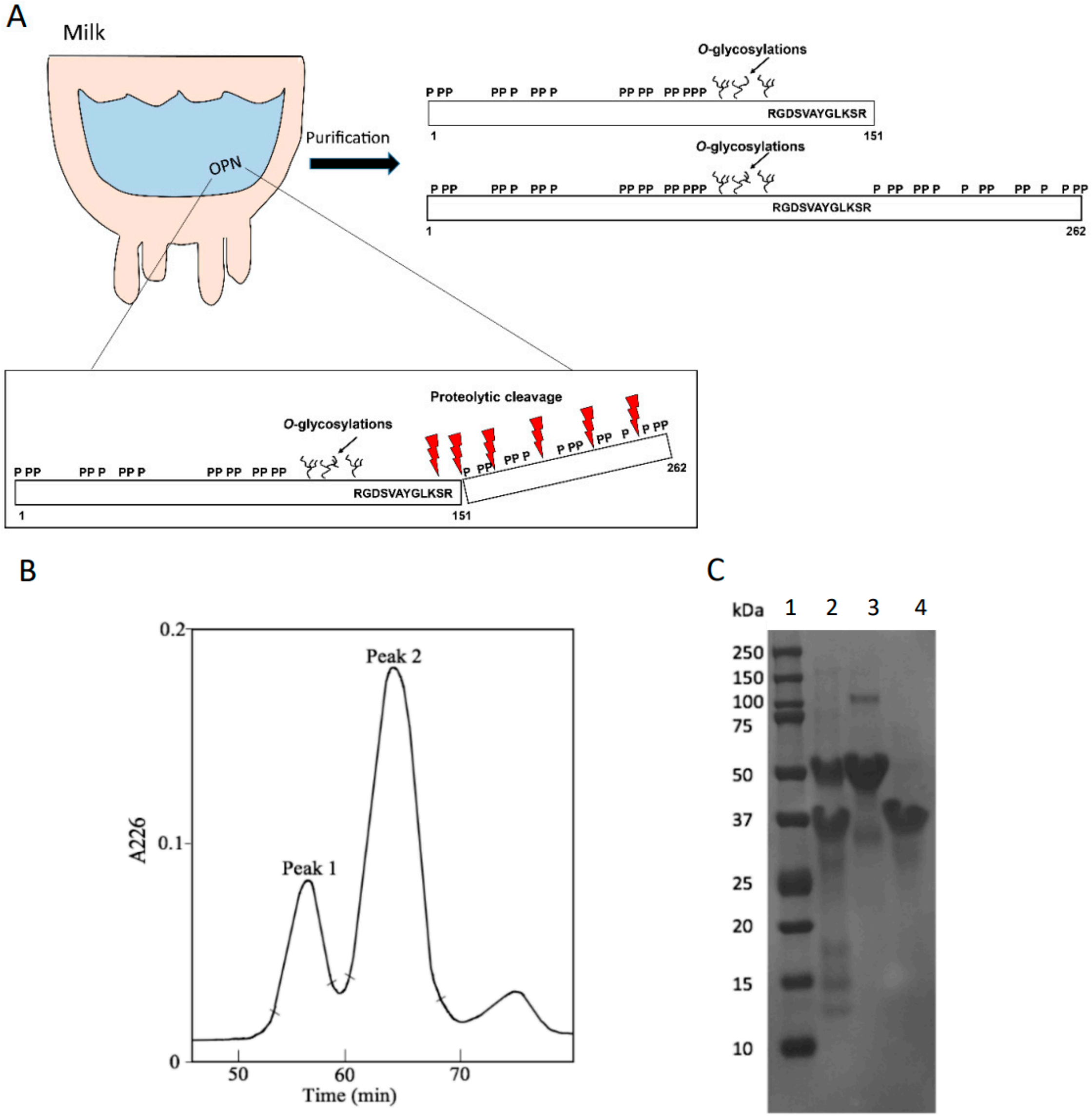

Figure S1. Separation of full-length OPN and N-terminal fragments present in OPN purified from bovine milk. (A) In milk, osteopontin (OPN) exists as a full-length protein and as N-terminal fragments generated by endogenous proteolytic cleavage. The C-terminal part of OPN is completely degraded. Full-length OPN and the N-terminal fragments are purified together from the milk. (B) Gel filtration of OPN from bovine milk on a Superdex 200 HR 10/30 column equilibrated in 0.1 M NH4HCO3, pH 8.1 at a flow of 0.4 ml/min. (C) The proteins were separated on a 14% Tris-glycine gel by SDS-PAGE and visualized by Coomassie Brilliant Blue staining. Lane 1, molecular weight standards; lane 2, OPN purified from bovine milk; lanes 3- 4, peak 1 and peak 2 from gel filtration of bovine milk OPN in (A).

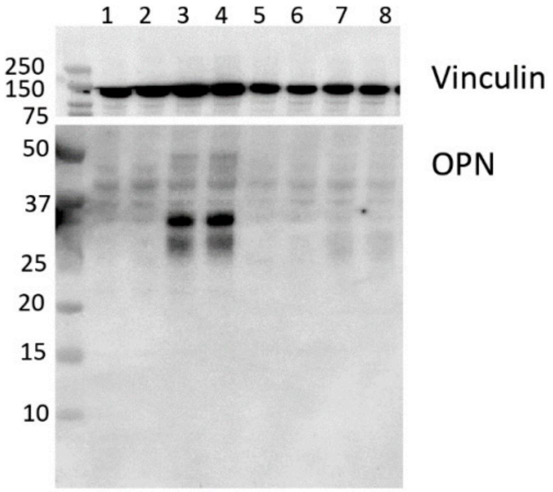

Figure S2. Evaluation of cell surface binding of OPN to Caco-2 cells by trypsin treatment. Caco-2 cells were incubated with OPN or serum-free medium (SFM) for 2h, washed with PBS, and then incubated with 0.05% trypsin for 3 min. before lysis using RIPA buffer. OPN was detected in the lysates by western blotting using the monoclonal antibody MAB193p. The intracellular protein vinculin was also detected as control for the extracellular activity of trypsin. Lane 1-2, SFM; Lanes 3-4, OPN; Lanes 5-6, SFM with trypsin; Lanes 7-8, OPN with trypsin.

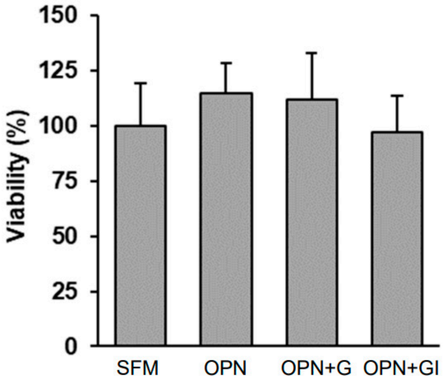

Figure S3. Cytotoxicity of OPN after simulated gastrointestinal digestion. Caco-2 cells were incubated with OPN (50 µg/ml) before and after gastric digestion (G) with pepsin, or further intestinal digestion with trypsin, chymotrypsin and elastase (GI). Incubation of the Caco-2 cells in serum-free medium (SFM) was used as control. The evaluation of cell viability was performed using a MTT assay. Data are expressed as mean ± SD (n = 10) and representative for three individual experiments.

A

Caco-2:HT29-MTX	P_{app} (cm/s)
100:0	$7.96 \pm 0.75 \cdot 10^{-7}$
90:10	$9.64 \pm 2.53 \cdot 10^{-7}$
75:25	$1.08 \pm 0.30 \cdot 10^{-6}$
0:100	$3.60 \pm 1.82 \cdot 10^{-6}$
No cells	$5.55 \pm 0.0094 \cdot 10^{-5}$

B

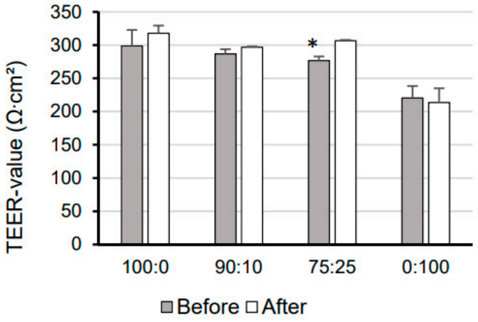

Figure S4. Apparent permeability coefficient and TEER of Caco-2/HT29-MTX co-cultures. (A) Apparent permeability coefficient (Papp) of Caco-2/HT29-MTX co-cultures grown and differentiated for 20 days on transwell membranes. Papp was determined from the flux of the paracellular marker mannitol. Values are presented as mean ± SD (n = 2). (B) TEER of Caco-2/HT29-MTX co-cultures before and after the transport assay. Caco-2:HT29- MTX proportions are given below bars. Values are presented as mean ± SD (n = 5-6 for "before", n = 2 for "after"). * denotes significant difference ($p < 0.05$) between values before and after the transport assay.
